# Association of Prenatal Exposure to Benzodiazepines With Development of Autism Spectrum and Attention-Deficit/Hyperactivity Disorders

**DOI:** 10.1001/jamanetworkopen.2022.43282

**Published:** 2022-11-22

**Authors:** Vincent Chin-Hung Chen, Shu-I Wu, Chiao-Fan Lin, Mong-Liang Lu, Yi-Lung Chen, Robert Stewart

**Affiliations:** 1 Department of Psychiatry, Chiayi Chang Gung Memorial Hospital, Chiayi, Taiwan; 2 Department of Psychiatry, Chang Gung University, Taoyuan, Taiwan; 3 Department of Medicine, Mackay Medical College, New Taipei City, Taiwan; 4 Department of Psychiatry, Mackay Memorial Hospital, Taipei, Taiwan; 5 Department of Psychiatry, Linkou Chang Gung Memorial Hospital, Taoyuan, Taiwan; 6 Department of Psychiatry, Wan-Fang Hospital & School of Medicine, College of Medicine, Taipei Medical University, Taipei, Taiwan; 7 Department of Healthcare Administration, Asia University, Taichung, Taiwan; 8 Department of Psychology, Asia University, Taichung, Taiwan; 9 Institute of Psychiatry, Psychology and Neuroscience, King’s College London, London, United Kindgom; 10 South London and Maudsley NHS Foundation Trust, London, United Kingdom

## Abstract

**Importance:**

Prenatal exposure to benzodiazepines is reported to be associated with neurodevelopmental disorders among children, but associations of maternal genetic confounding with neurodevelopmental disorders among children have not been taken into consideration.

**Objective:**

To ascertain whether prenatal benzodiazepine exposure was associated with development of autism spectrum disorder (ASD) and attention-deficit/hyperactivity disorder (ADHD).

**Design, Setting, and Participants:**

This cohort study used linked data from birth certificate registration and the Taiwan National Health Insurance Research Database from January 1, 2004, to December 31, 2017, on 1 138 732 mothers with 1 516 846 live births between January 1, 2004, and December 31, 2017. Data were analyzed between February 20, 2021, and September 19, 2022.

**Exposure:**

Benzodiazepine exposure during pregnancy (first trimester to third trimester) was defined as having at least one benzodiazepine prescription dispensed.

**Main Outcomes and Measures:**

The main outcomes were ADHD and ASD.

**Results:**

There were 1 516 846 children (mean [SD] gestational age, 38.5 [1.8] years; 789 455 boys [52.0%]) born full term who were younger than 14 years of age and followed up to 2017; 5.0% of the children (n = 76 411) were exposed to a benzodiazepine during pregnancy. Benzodiazepine exposure during pregnancy was associated with increased risks of ADHD (first trimester exposure: hazard ratio [HR], 1.24 [95% CI, 1.20-1.28]; second trimester exposure: HR, 1.27 [95% CI, 1.21-1.34]; third trimester exposure: HR, 1.25 [95% CI, 1.14-1.37]) and ASD (first trimester exposure: HR, 1.13 [95% CI, 1.05-1.21]; second trimester exposure: HR, 1.10 [95% CI, 0.98-1.22]; third trimester exposure: HR, 1.21 [95% CI, 1.00-1.47]). However, no differences were found with unexposed sibling controls during the same time frame for ADHD (first trimester exposure: HR, 0.91 [95% CI, 0.83-1.00]; second trimester exposure: HR, 0.89 [95% CI, 0.78-1.01]; third trimester exposure: HR, 1.08 [95% CI, 0.83-1.41]) or ASD (first trimester exposure: HR, 0.92 [95% CI, 0.75-1.14]; second trimester exposure: HR, 0.97 [95% CI, 0.71-1.33]; third trimester exposure: HR, 1.07 [95% CI, 0.53-2.16]). Similar findings were also noted in the stratification analysis of short-acting and long-acting benzodiazepines.

**Conclusions and Relevance:**

This cohort study suggests that previously described adverse neurodevelopmental outcomes associated with benzodiazepine exposure during pregnancy were likely to be accounted for by maternal genetic confounding.

## Key Points

**Question:**

Is exposure to benzodiazepines in utero associated with development of autism spectrum or attention-deficit/hyperactivity disorders?

**Findings:**

This cohort study of 1 516 846 children found that, after adjusting for possible confounders and accounting for possible parental genetic or familial factors, benzodiazepine exposure during pregnancy was not associated with increased risks of neurodevelopmental disorders.

**Meaning:**

Knowledge of outcomes among offspring associated with prenatal exposure to benzodiazepines can help inform their use during pregnancy.

## Teaser

This cohort study uses data from the Taiwan National Health Insurance Research Database to assess whether prenatal benzodiazepine exposure was associated with development of autism spectrum disorder (ASD) and attention-deficit/hyperactivity disorder (ADHD).

## Introduction

Approximately 10% to 30% of pregnant women experience mental disorders, including mood or anxiety spectrum disorders^1^ Benzodiazepine agents may be considered at this point to help ameliorate symptoms of anxiety or depression; the prevalence of prescribed benzodiazepine use during pregnancy has been estimated at 1.9% globally.^2^ However, the safety of these agents to the developing fetus and newborn has been called into question. In particular, benzodiazepines are able to cross the placenta, have been found to be present in amniotic fluid and breast milk, and have been listed in the US Food and Drug Administration pregnancy category of “potential benefits may warrant use of the drug in pregnant women despite potential human fetal risks” or “the risks outweigh potential benefits,” indicating potential harm to the fetus.^3^ Although influences on cortical development or neuronal networks have been described in rodents exposed to benzodiazepines during the first trimester of pregnancy,^4^ investigations of neurodevelopmental outcomes in humans, such as autism spectrum disorder (ASD) or attention-deficit/hyperactivity disorder (ADHD), have been scarce, to our knowledge. One cohort study found no significantly increased risks of ADHD symptoms or fine or greater motor deficits (weighted β, 0.67; 95% CI, 0.21-1.13).^5^ The authors proposed that residual confounding by indication (eg, the maternal depressive or anxiety disorder resulting in benzodiazepine use) might partially explain the observed moderately increased risks of communication and gross motor deficits. Untreated or inadequately treated maternal depressive and anxiety symptoms during pregnancy have been found to be associated with unmeasured genetic or familial factors or confounding by indication, and need to be accounted for when considering neurodevelopmental outcomes.^6^ The literature has reported that children whose mothers had depressive or anxiety symptoms during pregnancy had significantly higher risks of developing ADHD (odds ratio, 2.80 [95% CI, 2.20-3.57] comparing mothers with or without depression, a 19.2% [95% CI, 8.6%-31.0%] difference in numerous ADHD symptoms comparing mothers with or without anxiety during pregnancy)^7^ or ASD, (odds ratio, 1.72; [95% CI, 1.54-1.96] comparing mothers with or without depression).^8,9^ To our knowledge, no study has investigated ASD in this respect. Moreover, although few studies have examined how different trimesters of pregnancy may be associated with neurodevelopmental adverse outcomes,^10,11^ they did not control for potential maternal genetic confounding.

Given the current uncertainty, we assembled a population-based cohort study to ascertain whether there is an association between benzodiazepine exposure during different trimesters of pregnancy and the adverse neurodevelopmental outcomes of ADHD and ASD. Sibling controls, a parallel comparison by paternal exposure status, was also adopted to better control for maternal genetic confounding.

## Method

### Study Participants

All procedures involving human patients were approved by the research ethics committee of the China Medical University and Hospital. Patient informed consent is not required because this study used existing data of the Taiwan National Health Insurance Research Database, which are not individually identifiable. This study followed the Strengthening the Reporting of Observational Studies in Epidemiology (STROBE) reporting guideline. Data used in this cohort were drawn from the Taiwan National Health Insurance Research Database.^12^ All Taiwan residents are required to join the National Health Insurance program until death or emigration. We used the Taiwan birth certificate registration from January 1, 2004, to December 31, 2017, to ascertain all live-born children with full-term births (>37 weeks) and obtained information on their date of birth and gestational age. Birth certificate registration in Taiwan is compulsory for all health care facilities. Maternal exposure to prescribed benzodiazepine derivatives was defined as Anatomical Therapeutic Chemical Classification codes starting with N05BA, comprising diazepam, chlordiazepoxide, clonazepam, medazepam, oxazepam, lorazepam, bromazepam, alprazolam, nordazepam, fludiazepam, or clotiazepam; we further categorized benzodiazepines into long-acting benzodiazepines (diazepam, flurazepam, chlordiazepoxide, and clonazepam) and short-acting benzodiazepines (medazepam, oxazepam, lorazepam, bromazepam, alprazolam, nordazepam, fludiazepam, and clotiazepam).^13^ Prescription information was identified in dispensation records from the data sets of details of ambulatory care orders and expenditures for prescriptions dispensed at contracted pharmacies, which include all prescribed and filled medication dispensations and accompanying prescriptions prescribed by general practitioners and specialists for all outpatient visits from hospitals or community pharmacies. Linkage between these 2 data sets was thus used to assess maternal benzodiazepine exposure before and during pregnancy. Further linkage to the Taiwan Maternal and Child Health Database, including data on 99.8% of all births nationwide in Taiwan,^14^ enabled us to obtain complete information on children, their fathers, and paternal benzodiazepine exposure during the period from January 1, 2004, to December 31, 2014.

### Benzodiazepine Exposure in Different Trimesters of Pregnancy

**Figure 1.**
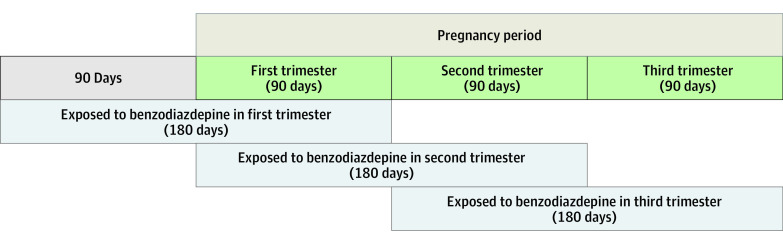
Overview of the Classification of Benzodiazepine Exposure During Pregnancy Pregnancy was split into periods before and after pregnancy. Three different trimesters are defined: first trimester, 90 days or less after estimated conception; second trimester, 91 to 180 days after estimated conception; and third trimester, 181 to 270 days after estimated conception. However, because medications for chronic disease in Taiwan are typically prescribed for at least 28 days and at most 90 days at a time, we extended an additional 90 days for the definitions of trimester: first trimester, 90 days before and after estimated conception; second trimester, 180 days or less after estimated conception; and third trimester, 90 to 270 days after estimated conception.

Trimesters were defined as the following time periods: (1) first trimester, 90 days or less after the estimated date of conception; (2) second trimester, 91 to 180 days after estimated conception; and (3) third trimester, 181 to 270 days after estimated conception. The date of conception was calculated using the date of delivery and the gestational age from maternity records, and the date information was recorded using the last menstrual period method. We followed a previous study conducted in Sweden^6^ by expanding an additional 90 days before each trimester to ascertain maternal benzodiazepine exposure because the medications for chronic disease in Taiwan, similar to Sweden, are typically prescribed for at least 28 days and at most 90 days at a time. Thus, medication taken around a trimester may have been prescribed 90 days before the start of the trimester. Figure 1 was used to visualize the classification of benzodiazepine exposure in different trimesters of pregnancy. Specifically, to compare the risks of maternal benzodiazepine exposures associated with outcomes of interest, we defined the following time periods for maternal benzodiazepine exposures during different trimesters: (1) benzodiazepine exposure during the first trimester (90 days before and after the estimated date of conception), (2) benzodiazepine exposure during the second trimester (0 to 180 days after estimated conception), and (3) benzodiazepine exposure during the third trimester (90 to 270 days after estimated conception).

### Outcome Measures

All children from pregnancies exposed to benzodiazepines and those unexposed to benzodiazepines were followed up from birth to the incident diagnoses of ADHD or ASD, death, emigration, or the end of the study in 2017. These diagnoses were ascertained on the basis of at least 1 inpatient record or at least 3 outpatient diagnoses based on the *International Classification of Diseases, Ninth Revision* (*ICD-9*) for ADHD (*ICD-9* code 314) and ASD (*ICD-9* code 299) and *International Statistical Classification of Diseases and Related Health Problems, Tenth Revision* (*ICD-10*) criteria for ADHD (*ICD-10* code F90) and ASD (*ICD-10* code F84). A previous study has suggested that using 3 to 4 instances of an outpatient diagnosis increases the validity of the diagnosis.^15^ The death status and time of death were determined from the Cause of Death data set.

### Covariates

Covariates were selected based on their relevance to children and outcomes and were categorized into child covariates (year of birth,^6^ gestational age, parity, low-income family status), maternal covariates (nationality [ie, Taiwanese or non-Taiwanese],^16^ age at the index birth, maternal smoking during pregnancy, maternal opioid drug use [Anatomical Therapeutic Chemical code N02A], and history of any mental disorders^17^ [*ICD-9* codes 290-319 and *ICD-10* codes F01-F99]), and paternal covariates (nationality, age at the index birth, and history of any mental disorders). Maternal and paternal covariates were considered time-varying covariates, and time before the conception date of each child was the covariate assessment period.

### Sensitivity Analysis

To evaluate possible indication bias, exposure misclassification, and detection of potential unmeasured confounding and indication bias, several sensitivity analyses were conducted. First, to address possible indication bias, we restricted our analysis to mothers with anxiety disorders or major depressive disorder. Second, to evaluate exposure misclassification, we used 3 different definitions of the medication dispensation window for trimester: (1) 30 days before and 90 days after the beginning of the trimester, (2) 90 days after the beginning of the trimester, and (3) prescription with days’ supply (eFigure in the Supplement). To examine further possible unmeasured familial confounding and indication bias, a negative control analysis was carried out using paternal benzodiazepine exposure during the different periods and a parallel comparison by paternal exposure status, and comparisons of risks for exposures before pregnancy. For negative control analysis, although paternal valproic acid exposure in mice might be associated with behavioral alterations in offspring through associations with germline epigenome in the animal model,^18^ no research to date has reported negative associations with child outcomes through epigenetic alterations from paternal benzodiazepine exposure, to our knowledge. Considering first trimester paternal benzodiazepine exposure, for example, children born to fathers with benzodiazepine dispensations during the first trimester period (90 days before and after the estimated date of conception) were selected and compared with unexposed children. Furthermore, sibling comparisons were also conducted to examine the association between paternal benzodiazepine exposure and neurobehavioral outcomes. Finally, to compare the timing of maternal use of benzodiazepines associated with the outcome before and around the time of pregnancy (eFigure in the Supplement), an adjusted population-wide and sibling comparison was conducted with the before-pregnancy group serving as the reference group.

### Statistical Analysis

Demographic characteristics were described for the whole sample and for subsamples with benzodiazepine exposures at different times during pregnancy, as well as unexposed children. Outcomes were first compared between pregnancy with or without maternal benzodiazepine exposures using population-wide comparison and sibling comparisons. The population-wide comparison group comprised children born to mothers without any benzodiazepine dispensation during pregnancy. Cox proportional hazards regression analysis (using age in days as a timescale) was used to estimate risks for the 2 neurobehavioral outcomes of ASD or ADHD. Robust SEs were used in regression models to account for clustering of siblings from the same biological mother. For adjusted associations, we controlled for year of birth, maternal and paternal nationality, gestational age, parity, maternal smoking during pregnancy, maternal opioid use, parental age at childbirth, and parental history of mental disorders.

For sibling comparisons, we conducted within-family analysis to address potential maternal genetic confounding by using conditional Cox proportional hazards regression with the biological mother as the stratum variable to compare risks of neurobehavioral outcomes between exposed and unexposed siblings, and adjusted for year of birth, gestational age, parity, maternal smoking during pregnancy, maternal opioid use, parental age at childbirth, and parental history of mental disorders.

To examine whether the association between maternal benzodiazepine exposures during pregnancy and child neurodevelopmental disorders differed between short-acting and long-acting benzodiazepines, stratification analysis was conducted. We used SAS, version 9.4 (SAS Institute Inc) for statistical analyses with 95% CIs calculated based on 2-sided hypothesis testing. Data were analyzed between February 20, 2021, and September 19, 2022.

## Results

There were 1 547 702 children with full-term birth before 2014, and 30 856 were removed because their paternal age information was missing. The study cohort comprised 1 516 846 children younger than 14 years of age (789 455 boys [52.0%]), and 1 138 732 biological mothers and 1134 320 fathers; and 5.0% of the children (n = 76 411) were exposed to a benzodiazepine during pregnancy. Specifically, 4.6% (n = 70 451), 2.2% (n = 33 123), and 0.6% (n = 8828) were exposed to benzodiazepines during the first, second, and third trimesters of pregnancy, respectively (Table 1). The mean (SD) follow-up time (by the end of 2017) for the full child sample was 8.5 (3.2) years. Incidences of ASD (826 of 76 411 [1.1%] vs 13 276 of 1 440 435 [0.9%]) and ADHD (3722 of 76 411 [4.9%] vs 55 556 of 1 440 435 [3.9%]), as well as the proportions with low-income family status (6497 of 76 411 [8.5%] vs 87 541 of 1 440 435 [6.1%]), maternal opioid use (6364 of 76 411 [8.3%] vs 62 630 of 1 440 435 [4.3%]), smoking during pregnancy (45 of 76 411 [0.1%] vs 546 of 1 440 435 [0.04%]), and maternal (24 824 of 76 411 [32.5%] vs 123 102 of 1 440 435 [8.5%]) and paternal mental disorders (9391 of 76 411 [12.3%] vs 128 186 of 1 440 435 [8.9%]), were all higher among the benzodiazepine-exposed children than those unexposed.

**Table 1.** Descriptive Statistics for Sample by Exposure to Benzodiazepine During Pregnancy^a^

Variable	Any exposure (n = 76 411)				Unexposed (n = 1 440 435)
	All trimesters (n = 76 411)	First trimester (n = 70 451)	Second trimester (n = 33 123)	Third trimester (n = 8828)	
**Child covariate**					
Parity					
1	47 223 (61.8)	43 710 (62.0)	20 504 (61.9)	5213 (59.1)	863 322 (59.9)
2	24 839 (32.5)	22 786 (32.3)	10 703 (32.3)	2994 (33.9)	501 183 (34.8)
≥3	4349 (5.7)	3955 (5.6)	1916 (5.8)	621 (7.0)	75 930 (5.3)
Low-income family	6497 (8.5)	5857 (8.3)	3126 (9.4)	1024 (11.6)	87 541 (6.1)
Gestational age, mean (SD), wk	38.5 (1.9)	38.4 (1.9)	38.5 (2.0)	38.4 (2.2)	38.5 (1.8)
Child age, mean (SD), y	8.4 (3.2)	8.4 (3.2)	8.4 (3.3)	8.6 (3.2)	8.0 (3.2)
Child diagnosis					
Autism spectrum disorder	826 (1.1)	754 (1.1)	345 (1.0)	103 (1.2)	13 276 (0.9)
Attention-deficit/hyperactivity disorder	3722 (4.9)	3430 (4.9)	1658 (5.0)	440 (5.0)	55 556 (3.9)
**Pregnancy covariate**					
Maternal covariate					
Age at birth, y					
<20	791 (1.0)	3430 (4.9)	1658 (5.0)	440 (5.0)	13 564 (0.9)
20-24	8863 (11.6)	8074 (11.5)	4004 (12.1)	1087 (12.3)	151 057 (10.5)
25-29	24 643 (32.3)	22 738 (32.3)	10 736 (32.4)	2763 (31.3)	460 678 (32.0)
30-34	28 260 (37.0)	26 111 (37.1)	11 843 (35.8)	3212 (36.4)	567 482 (39.4)
35-39	11 948 (15.6)	11 081 (15.7)	5246 (15.8)	1398 (15.8)	217 349 (15.1)
≥40	1906 (2.5)	1753 (2.5)	919 (2.8)	244 (2.8)	30 305 (2.1)
Taiwanese nationality	25 241 (33.0)	23 074 (32.8)	10 957 (33.1)	3060 (34.7)	473 451 (32.9)
Opioid use	6364 (8.3)	5978 (8.5)	2723 (8.2)	678 (7.7)	62 630 (4.3)
Smoking during pregnancy	45 (0.1)	40 (0.1)	25 (0.1)	10 (0.1)	546 (0.04)
Anxiety disorders or major depressive disorder	8963 (11.7)	8492 (12.1)	4785 (14.4)	1334 (15.1)	30 832 (2.1)
Any mental disorders	24 824 (32.5)	23 365 (33.2)	12 042 (36.4)	3143 (35.6)	123 102 (8.5)
Benzodiazepine use					
Short-acting	60 622 (79.3)	46 368 (65.8)	19 720 (59.5)	5070 (57.4)	NA
Long-acting	50 149 (65.6)	54 683 (77.6)	27 034 (81.6)	8737 (99.0)	NA
Paternal covariate					
Age at birth, y					
<20	155 (0.2)	129 (0.2)	68 (0.2)	29 (0.3)	2559 (0.2)
20-24	3433 (4.5)	3142 (4.5)	1572 (4.7)	422 (4.8)	53 308 (3.7)
25-29	16 113 (21.1)	14 865 (21.1)	7076 (21.4)	1782 (20.2)	288 818 (20.1)
30-34	28 748 (37.6)	26 512 (37.6)	12 126 (36.6)	3243 (36.7)	569 273 (39.5)
35-39	18 993 (24.9)	17 568 (24.9)	8137 (24.6)	2160 (24.5)	371 697 (25.8)
≥40	8969 (11.7)	8235 (11.7)	4144 (12.5)	1192 (13.5)	154 780 (10.7)
Taiwanese nationality	23 173 (30.3)	21 203 (30.1)	9990 (30.2)	2791 (31.6)	409 958 (28.5)
Any mental disorders	9391 (12.3)	8671 (12.3)	4362 (13.2)	1333 (15.1)	128 186 (8.9)

Abbreviation: NA, not applicable.

^a^ Data are presented as number (percentage) unless otherwise stated.

**Figure 2.**
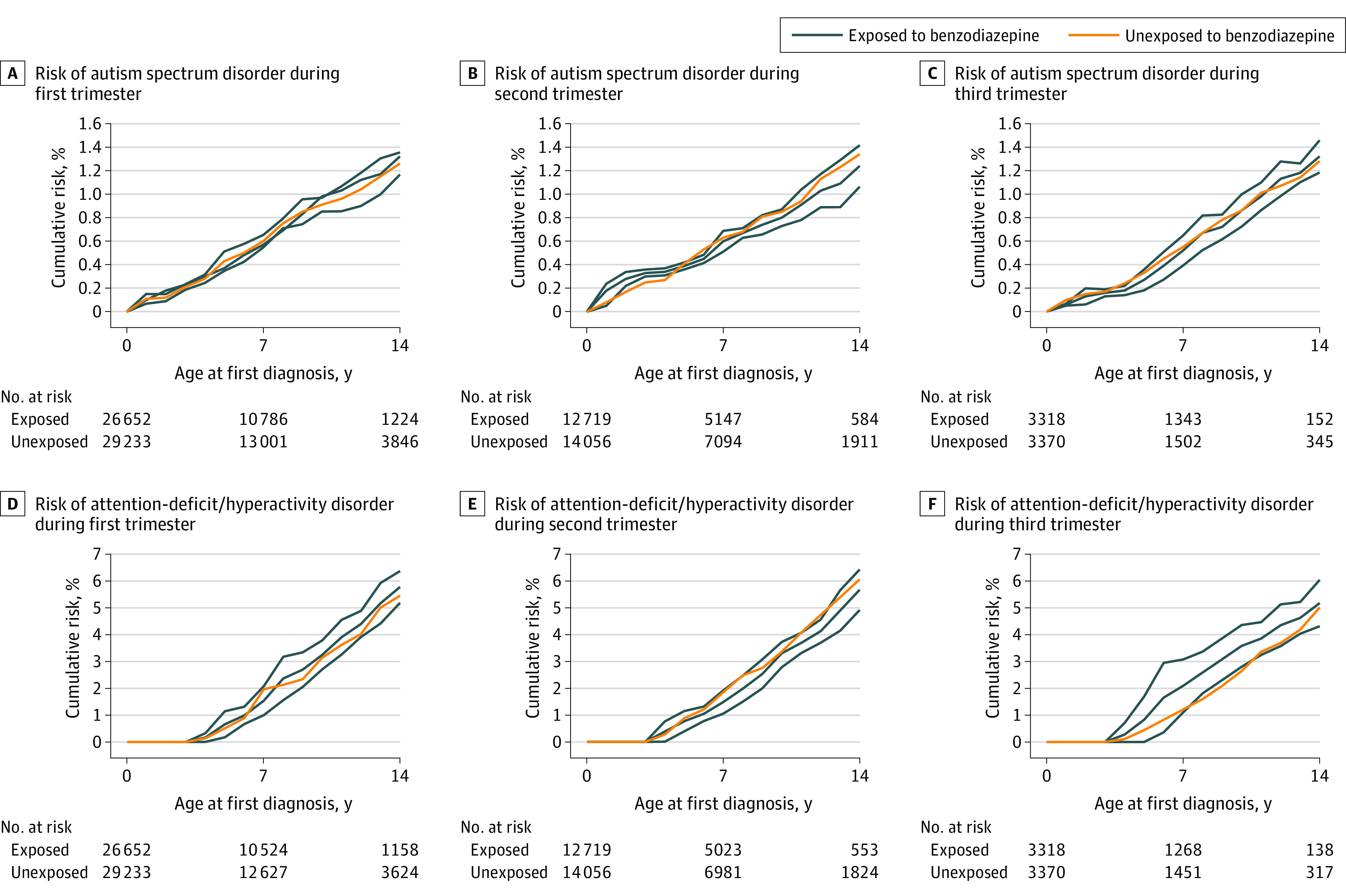
Risk of Autism Spectrum Disorder and Attention-Deficit/Hyperactivity Disorder by Siblings Discordant for Maternal Benzodiazepine Exposure During the First, Second, and Third Trimesters The upper and lower blue lines in each panel indicate pointwise 95% CIs for patients exposed to benzodiazepines.

Table 2 describes the occurrence of neurodevelopmental disorders according to maternal benzodiazepine exposure during the first, second, and third trimesters. These analyses showed higher risks of ASD (first trimester exposure: hazard ratio [HR], 1.13 [95% CI, 1.05-1.21]; second trimester exposure: HR, 1.10 [95% CI, 0.98-1.22]; third trimester exposure: HR, 1.21 [95% CI, 1.00-1.47]) and ADHD (first trimester exposure: HR, 1.24 [95% CI, 1.20-1.28]; second trimester exposure: HR, 1.27 [95% CI, 1.21-1.34]; third trimester exposure: HR, 1.25 [95% CI, 1.14-1.37]) among children exposed to benzodiazepines in unadjusted analyses for most exposure periods, as well as a higher risk of ADHD associated with first and second trimester benzodiazepine exposure in adjusted analyses (first trimester exposure: HR, 1.06 [95% CI, 1.02-1.10]; second trimester exposure: HR, 1.07 [95% CI, 1.02-1.12]). However, when we used the sibling comparison models to address potential maternal genetic confounding, for all time periods during pregnancy there were no significant associations of benzodiazepine exposure with ADHD (first trimester exposure: HR, 0.91 [95% CI, 0.83-1.00]; second trimester exposure: HR, 0.89 [95% CI, 0.78-1.01]; third trimester exposure: HR, 1.08 [95% CI, 0.83-1.41]) or ASD (first trimester exposure: HR, 0.92 [95% CI, 0.75-1.14]; second trimester exposure: HR, 0.97 [95% CI, 0.71-1.33]; third trimester exposure: HR, 1.07 [95% CI, 0.53-2.16]) (Figure 2). To address potential indication bias, we restricted our analysis to mothers with anxiety disorders or major depressive disorder, and the results remained the same (eTable 1 in the Supplement). Considering possible exposure misclassification of maternal benzodiazepine dispensation, we used 3 different exposure time windows: (1) 30 days before and 90 days after the beginning of trimester, (2) 90 days after the beginning of trimester, and (3) prescription with days’ supply; all results were still consistent with the main analyses (eTable 2 in the Supplement). Finally, we observed that associations between maternal benzodiazepine exposure during the first, second, and third trimesters of pregnancy and child neurodevelopmental outcomes were consistent with short-acting and long-acting benzodiazepines (Table 3).

**Table 2.** Associations of Neurodevelopmental Disorders With Maternal Benzodiazepine Exposures During Pregnancy Using Population-Wide and Sibling Comparison Models

Period	Hazard ratio (95% CI)		
	Crude model	Adjustment model^a^	Sibling comparison model^b^
First trimester			
Autism spectrum disorder	1.13 (1.05-1.21)	0.96 (0.89-1.03)	0.92 (0.75-1.14)
Attention-deficit/hyperactivity disorder	1.24 (1.20-1.28)	1.06 (1.02-1.10)	0.91 (0.83-1.00)
Second trimester			
Autism spectrum disorder	1.10 (0.98-1.22)	0.91 (0.82-1.02)	0.97 (0.71-1.33)
Attention-deficit/hyperactivity disorder	1.27 (1.21-1.34)	1.07 (1.02-1.12)	0.89 (0.78-1.01)
Third trimester			
Autism spectrum disorder	1.21 (1.00-1.47)	1.01 (0.83-1.23)	1.07 (0.53-2.16)
Attention-deficit/hyperactivity disorder	1.25 (1.14-1.37)	1.05 (0.95-1.15)	1.08 (0.83-1.41)

^a^ Adjusted model controlled for year of birth, parity, gestational age, low-income family status, paternal and maternal nationality, paternal and maternal age at child birth, paternal and maternal history of mental disorders, maternal smoking during pregnancy, and maternal opioid use.

^b^ Sibling comparison controlled for year of birth, parity, gestational age, paternal and maternal age at child birth, paternal and maternal history of mental disorders, maternal smoking during pregnancy, and maternal opioid use. For analysis of sibling comparison, there were 55 885 children comprising 26 334 discordant pairs of differentially exposed siblings (26 652 exposed and 29 233 unexposed) for the first trimester, 26 775 children comprising 12 605 discordant pairs of differentially exposed siblings (12 719 exposed and 14 056 unexposed) for the second trimester, and 7018 children comprising 3279 discordant pairs of differentially exposed siblings (3318 exposed and 3700 unexposed) for the third trimester.

**Table 3.** Associations of Neurodevelopmental Disorders With Maternal Benzodiazepine Exposures During Pregnancy Using Population-Wide and Sibling Comparison Models, Stratified by Short-Acting and Long-Acting Benzodiazepines

Period	Hazard ratio (95% CI)			
	Short-acting benzodiazepine		Long-acting benzodiazepine	
	Adjustment model^a^	Sibling comparison^b^	Adjustment model^a^	Sibling comparison^b^
First trimester				
Autism spectrum disorder	1.07 (0.99-1.16)	1.03 (0.80-1.31)	0.94 (0.86-1.03)	0.77 (0.58-1.01)
Attention-deficit/hyperactivity disorder	1.12 (1.08-1.17)	0.93 (0.83-1.04)	1.12 (1.07-1.17)	0.97 (0.86-1.09)
Second trimester				
Autism spectrum disorder	0.99 (0.88-1.10)	1.16 (0.82-1.63)	0.85 (0.74-0.99)	1.00 (0.62-1.61)
Attention-deficit/hyperactivity disorder	1.12 (1.08-1.17)	0.88 (0.75-1.02)	1.12 (1.07-1.17)	0.87 (0.73-1.03)
Third trimester				
Autism spectrum disorder	1.13 (1.07-1.19)	1.11 (0.57-2.17)	1.13 (1.06-1.20)	2.45 (0.38-15.71)
Attention-deficit/hyperactivity disorder	1.22 (1.12-1.33)	1.03 (0.78-1.37)	1.21 (1.08-1.36)	1.01 (0.70-1.47)

^a^ Adjusted model controlled for year of birth, parity, gestational age, low-income family status, paternal and maternal nationality, paternal and maternal age at child birth, paternal and maternal history of mental disorders, maternal smoking during pregnancy, and maternal opioid use.

^b^ Sibling comparison controlled for year of birth, parity, gestational age, paternal and maternal age at child birth, paternal and maternal history of mental disorders, maternal smoking during pregnancy, and maternal opioid use. For analysis of sibling comparison of short-acting benzodiazepine exposure, there were 37 904 children comprising 17 780 discordant pairs of differentially exposed siblings (18 125 exposed and 19 779 unexposed) for the first trimester, 19 252 children comprising 9022 discordant pairs of differentially exposed siblings (9181 exposed and 10 071 unexposed) for the second trimester, and 5796 children comprising 2705 discordant pairs of differentially exposed siblings (2777 exposed and 3019 unexposed) for the third trimester. For analysis of sibling comparison of long-acting benzodiazepine exposure, there were 34 452 children comprising 16 202 discordant pairs of differentially exposed siblings (16 481 exposed and 17 971 unexposed) for the first trimester, 15 009 children comprising 7054 discordant pairs of differentially exposed siblings (7158 exposed and 7851 unexposed) for the second trimester, and 3572 children comprising 1656 discordant pairs of differentially exposed siblings (1702 exposed and 1870 unexposed) for the third trimester.

For detecting potential unmeasured confounding, we investigated paternal benzodiazepine dispensations at different timings (eTable 3 in the Supplement). Weak but significant associations were found with ADHD for first (HR, 1.03 [95% CI, 1.00-1.08]), second (HR, 1.03 [95% CI, 1.00-1.08]), and third trimester (HR, 1.04 [95% CI, 1.00-1.08]) benzodiazepine exposures and with ASD for third trimester (HR, 1.04 [95% CI, 1.00-1.08]) benzodiazepine exposures in adjusted models, but most associations were not observed (except for third trimester benzodiazepine exposures for ASD; HR, 1.31 [95% CI, 1.02-1.69]) using sibling comparison models.

When comparing maternal benzodiazepine exposure before (eTable 4 in the Supplement) and during pregnancy, we did not observe any significant association between maternal benzodiazepine exposure and neurodevelopmental disorder occurrence during any trimester in the adjusted population-wide comparison or the sibling comparison (eTable 5 in the Supplement).

## Discussion

To our knowledge, this large population-based cohort study is the first to apply a sibling comparison method to examine the association between benzodiazepine exposures during pregnancy and subsequent neurodevelopmental disorders. Our key finding was that, although benzodiazepine exposure during pregnancy may be associated with increased risks of ADHD or ASD when compared with nonexposed population-wide controls, no significant increase in such risks was found when compared with sibling controls. Taken together, these results suggest that associations with in utero benzodiazepine exposure may be confounded by other maternal genetic or environmental factors. This finding was further supported by the insignificant associations between ADHD and benzodiazepine exposures before pregnancy, as well as paternal benzodiazepine use during the index pregnancy when compared with paternal sibling controls.

Our findings of higher risks of ADHD and ASD among offspring associated with maternal benzodiazepine exposures using population-based controls were in contrast with a previous cohort study that reported no excess risk of ADHD.^5^ However, Lupattelli et al^5^ also proposed that residual confounding by indication may not be ruled out. With our sibling comparison, we were able to reduce such residual confounding and discovered consistent findings of no increased risk of ADHD or ASD associated with any benzodiazepine exposure timing. To our knowledge, no prior studies have examined such an association using a sibling control method. The discordance in results between population and sibling comparisons suggests that maternal psychological or physical conditions may be potential unmeasured confounders for poorer neurodevelopmental outcomes among offspring. These conditions might include other maternal anxiety and sleep problems recognized to be associated with neurodevelopmental problems among children.^7,19^ Instead of assuming risks of neurodevelopmental disorders as a direct consequence of intrauterine benzodiazepine exposure, it may be more important to reduce these neurodevelopmental problems by identifying mothers at risk and opportune windows for early interventions to prevent subsequent neurodevelopmental comorbidities.

We found no significant differences in the risks of ADHD and ASD for differently exposed children born from the same mother, regardless of whether they were exposed to short-acting or long-acting benzodiazepines. In addition, the slightly increased risk of ADHD found among children born to fathers with benzodiazepine dispensations during the index pregnancy, which was supported by our negative control analysis, also suggests either underlying heritability or familial confounders.^20,21^ Familial or shared environmental factors, such as peripartum sociodemographic or psychological conditions, nutrition, family climate, or social support, may also play important roles in the cause of ASD or ADHD.^21,22^ Hence, improvements in the prenatal mental health conditions of parents or primary caregivers for these children, as well as adequate medical or social management for individuals at risk, should at least be considered as potential means to modify risk for these neurobehavioral disorders among offspring.

### Limitations

This study has some limitations. First, we used information on benzodiazepine prescriptions to define the exposure status, and registered data may be subject to misclassification owing to nonadherence or nonuse of the prescription; however, we think it most likely that this was a nondifferential misclassification and will have been biased toward the null.^23^ Furthermore, we conducted an additional sensitivity analysis using different time periods during pregnancy resulting in similar findings, suggesting that our results were robust. Second, we cannot exclude further unmeasured confounding factors that were not included in the Taiwan National Health Insurance Research Database, such as lifestyle factors or illicit substance use. Similarly, although the application of sibling comparisons may have controlled for time-invariant maternal factors, such a design would not be able to control for unmeasured factors between different pregnancies. Third, no validation study for ADHD and ASD has been conducted with the Taiwan National Health Insurance Research Database, to our knowledge. However, this limitation might have a limited association with outcomes because it has been reported that the diagnostic accuracy for ADHD and ASD was higher than for the other child psychiatric disorders^24^; moreover, we also used multiple records of ADHD and ASD, which have been reported to be able to increase the accuracy of diagnosis.^15^ Fourth, we did not include a dose-response analysis because duration and timing of pregnancy are not easily disentangled, and the association of cumulative exposure with neurodevelopmental outcomes may depend on their timing; future studies can use an advanced statistical method, such as the weighted cumulative exposure model,^25^ to examine the potential complex time-varying association. Fifth, the Maternal and Child Health Database was established in 2004 and we included only children born before 2014 and followed them up to 2017. The age range for the children in our study was limited to 3 to 13 years, and that age range might be associated with the diagnosis of ADHD. Specifically, children younger than 4 years may be difficult to diagnose, and our study could not capture adolescent and adult ADHD. Sixth, the use of sibling comparison needs sufficient power,^26^ and although this was a large national study with about 1.5 million children, sibling comparison design shrank the sample size by excluding children who did not have discordant sibling pairs. Sibling comparison design shrank the sample size, especially for maternal benzodiazepine exposure during the third trimester, because the sample size in that trimester was significantly smaller than those in the first and second trimesters, which was also reflected in the comparatively wider 95% CI during the third trimester. Seventh, the generalizability of our results to other countries or different health care systems would need to be established.

## Conclusions

After adjusting for possible confounders and accounting for possible parental genetic or wider familial factors, this cohort study found no evidence that benzodiazepine exposure during pregnancy was associated with increased risks of ASD or ADHD among offspring. Our results challenge current assumptions of a potential association of neurodevelopmental disorders with maternal benzodiazepine use before or during pregnancy. Better identification of maternal mental health concerns, as well as possible interventions or provisions of guidance to build better nurturing and raising environments for newborns at risk, may be relevant to the prevention of adverse outcomes of neurodevelopmental disorders.

## Notes

Supplement. **eFigure.** Overview of the Classification of BZD Exposure Before Pregnancy and the Prescription With Days’ Supply During Pregnancy **eTable 1.** Associations of Neurodevelopmental Disorders With Maternal Benzodiazepine Exposures During the First, Second, and Third Trimester of Pregnancy Among Mothers With Anxiety Disorders or Depression Using Population-Wide and Sibling Comparison Models **eTable 2.** Sibling-Comparison Associations Between Maternal BZD During the First, Second, and Third Trimester of Pregnancy and Offspring Neurodevelopmental Outcomes Applying Different Definitions of BZD Exposure Duration **eTable 3.** Associations of Neurodevelopmental Disorders With Paternal BZD Dispensations During the First, Second, and Third Trimester of Pregnancy Using Population-Wide and Sibling Comparison Models **eTable 4.** Descriptive Statistics for Sample by Exposure to BZD Before Pregnancy **eTable 5.** Associations of Neurodevelopmental Disorders With Maternal Benzodiazepine Exposures During the First, Second, and Third Trimester of Pregnancy, Compared With Exposure Before Pregnancy Using Population-Wide and Sibling Comparison Models
