# Supplementary material for: Association of Prenatal Exposure to Benzodiazepines With Development of Autism Spectrum and Attention-Deficit/Hyperactivity Disorders
Source: JAMA Netw Open. 2022 Nov 22;5(11):e2243282. doi: 10.1001/jamanetworkopen.2022.43282 (PMC9682429; doi:10.1001/jamanetworkopen.2022.43282)
Supplement: Supplement. — eFigure. Overview of the Classification of BZD Exposure Before Pregnancy and the Prescription With Days’ Supply During Pregnancy eTable 1. Associations of Neurodevelopmental Disorders With Maternal Benzodiazepine Exposures During the First, Second, and Third Trimester of Pregnancy Among Mothers With Anxiety Disorders or Depression Using Population-Wide and Sibling Comparison Models eTable 2. Sibling-Comparison Associations Between Maternal BZD During the First, Second, and Third Trimester of Pregnancy and Offspring Neurodevelopmental Outcomes Applying Different Definitions of BZD Exposure Duration eTable 3. Associations of Neurodevelopmental Disorders With Paternal BZD Dispensations During the First, Second, and Third Trimester of Pregnancy Using Population-Wide and Sibling Comparison Models eTable 4. Descriptive Statistics for Sample by Exposure to BZD Before Pregnancy eTable 5. Associations of Neurodevelopmental Disorders With Maternal Benzodiazepine Exposures During the First, Second, and Third Trimester of Pregnancy, Compared With Exposure Before Pregnancy Using Population-Wide and Sibling Comparison Models [file jamanetwopen-e2243282-s001.pdf]

Supplementary Online Content

Chen VCH, Wu SI, Lin CF, Lu ML, Chen YL, Stewart R. Association of prenatal exposure to benzodiazepines with development of autism spectrum and attention-deficit/hyperactivity disorders. *JAMA Netw Open.* 2022;5(11):e2243282. doi:10.1001/jamanetworkopen.2022.43282

eFigure. Overview of the Classification of BZD Exposure Before Pregnancy and the Prescription With Days' Supply During Pregnancy
eTable 1. Associations of Neurodevelopmental Disorders With Maternal Benzodiazepine Exposures During the First, Second, and Third Trimester of Pregnancy Among Mothers With Anxiety Disorders or Depression Using Population-Wide and Sibling Comparison Models
eTable 2. Sibling-Comparison Associations Between Maternal BZD During the First, Second, and Third Trimester of Pregnancy and Offspring Neurodevelopmental Outcomes Applying Different Definitions of BZD Exposure Duration
eTable 3. Associations of Neurodevelopmental Disorders With Paternal BZD Dispensations During the First, Second, and Third Trimester of Pregnancy Using Population-Wide and Sibling Comparison Models
eTable 4. Descriptive Statistics for Sample by Exposure to BZD Before Pregnancy
eTable 5. Associations of Neurodevelopmental Disorders With Maternal Benzodiazepine Exposures During the First, Second, and Third Trimester of Pregnancy, Compared With Exposure Before Pregnancy Using Population-Wide and Sibling Comparison Models

This supplementary material has been provided by the authors to give readers additional information about their work.

© 2022 Chen VCH et al. *JAMA Network Open.*

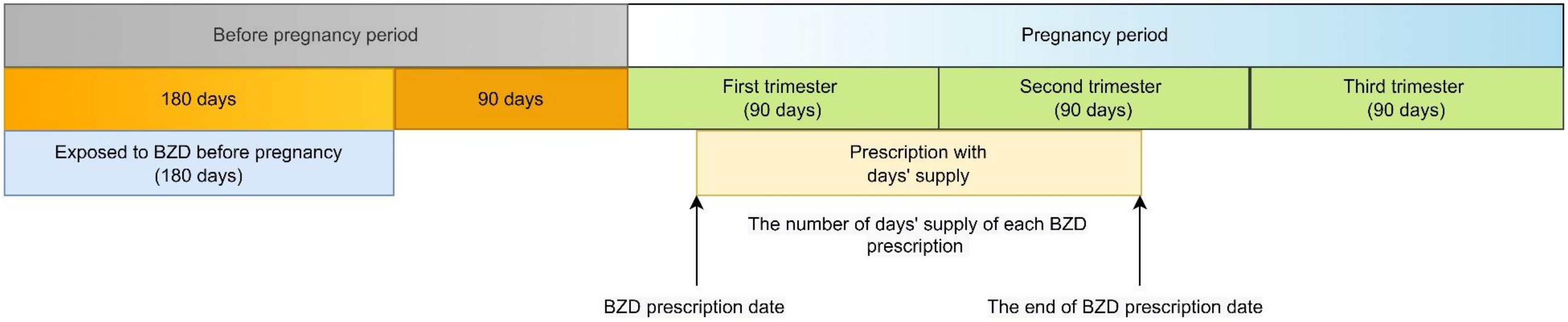

eFigure. Overview of the Classification of BZD Exposure Before Pregnancy and the Prescription With Days' Supply During Pregnancy

The pregnancy was split into before and after pregnancy periods. For BZD exposure before pregnancy, if the individual's windows period of the prescription date of BZD is 270 to 90 days before conception, one is defined as BZD exposure before pregnancy periods. For prescription with days' supply during pregnancy, three different trimesters are defined as first (0 to 90 days after conception), second (90 to 180 days after conception), and third (180 to 270 days after conception). If the individual's windows period of the prescription with days' supply (the date of BZD prescription date plus the number of days' supply) overlaps with any trimester windows, ones is defined as BZD exposure. In the example of the eFigure, the individual is exposed to BZD in first and second trimesters.

© 2022 Chen VCH et al. *JAMA Network Open.*

eTable 1. Associations of Neurodevelopmental Disorders With Maternal Benzodiazepine Exposures During the First, Second, and Third Trimester of Pregnancy Among Mothers With Anxiety Disorders or Depression Using Population-Wide and Sibling Comparison Models

	Models	
	Adjustment Model[a]	Sibling comparison[b]
Period	HR (95% CI)	HR (95% CI)
First Trimester		
Autism spectrum disorder	0.72 (0.60-0.87)	0.62 (0.25-1.54)
Attention-deficit/hyperactivity disorder	1.02 (0.93-1.12)	1.11 (0.79-1.55)
Second Trimester		
Autism spectrum disorder	0.60 (0.46-0.78)	0.24 (0.05-1.05)
Attention-deficit/hyperactivity disorder	0.99 (0.88-1.12)	0.95 (0.60-1.48)
Third Trimester		
Autism spectrum disorder	0.78 (0.51-1.19)	1.00 (0.91-1.11)
Attention-deficit/hyperactivity disorder	0.89 (0.71-1.12)	0.33 (0.08-1.30)

Abbreviations: CI, confidence interval, HR, hazard ratio.

[a]Adjusted model controlled for year of birth, parity, gestational age, low-income family status, maternal and paternal nationality, paternal and maternal age at child birth, paternal and maternal history of mental disorders, smoking during pregnancy, and maternal opioid drug use.

[b]Sibling comparison controlled for year of birth, parity, gestational age, paternal and maternal age at child birth, paternal and maternal history of mental disorders, maternal smoking during pregnancy, and maternal opioid drug use.

For analysis of sibling comparison, there were 3374 children consisted of 1609 discordant pairs of differentially exposed siblings (1649 were exposed and 1725 were unexposed) for first trimester, 2055 children consisted of 982 discordant pairs of differentially exposed siblings (992 were exposed and 1063 were unexposed) for second trimester. and 502 children consisted of 237 discordant pairs of differentially exposed siblings (243 were exposed and 259 were unexposed) for third trimester.

© 2022 Chen VCH et al. *JAMA Network Open.*

eTable 2. Sibling-Comparison Associations Between Maternal BZD During the First, Second, and Third Trimester of Pregnancy and Offspring Neurodevelopmental Outcomes Applying Different Definitions of BZD Exposure Duration

Period	30 days before and 90 days after the beginning of trimester	90 days after the beginning of trimester	Prescription with days' supply
	HR (95% CI)	HR (95% CI)	HR (95% CI)
First Trimester			
Autism spectrum disorder	0.90 (0.67-1.21)	0.87 (0.62-1.23)	0.90 (0.72-1.14)
Attention-deficit/hyperactivity disorder	0.91 (0.80-1.04)	0.86 (0.75-0.99)	0.89 (0.80-0.98)
Second Trimester			
Autism spectrum disorder	0.95 (0.43-2.13)	1.71 (0.68-4.27)	1.03 (0.88-1.21)
Attention-deficit/hyperactivity disorder	0.94 (0.68-1.29)	1.23 (0.89-1.70)	1.01 (0.92-1.11)
Third Trimester			
Autism spectrum disorder	0.87 (0.14-5.32)	0.29 (0.06-1.36)	0.61 (0.31-1.18)
Attention-deficit/hyperactivity disorder	1.30 (0.83-2.04)	1.04 (0.68-1.59)	1.03 (0.64-1.65)

Abbreviations: CI, confidence interval, HR, hazard ratio.

[a]Adjusted model controlled for year of birth, parity, gestational age, low-income family status, maternal and paternal nationality, paternal and maternal age at child birth, paternal and maternal history of mental disorders, smoking during pregnancy, and maternal opioid drug use.

[b]Sibling comparison controlled for year of birth, parity, gestational age, paternal and maternal age at child birth, paternal and maternal history of mental disorders, maternal smoking during pregnancy, and maternal opioid drug use.

For 30 days before and 30 days after conception, there were 28067 children consisted of 13260 discordant pairs of differentially exposed siblings (13359 were exposed and 14708 were unexposed) for first trimester, 4814 children consisted of 2256 discordant pairs of differentially exposed siblings (2272 were exposed and 2542 were unexposed) for second trimester and 2729 children consisted of 1272 discordant pairs of differentially exposed siblings (1282 were exposed and 1447 were unexposed).

© 2022 Chen VCH et al. *JAMA Network Open.*

For 90 days after conception, there were 23811 children consisted of 11224 discordant pairs of differentially exposed siblings (11303 were exposed and 12508 were unexposed) for first trimester, 4610 children consisted of 2147 discordant pairs of differentially exposed siblings (2170 were exposed and 2440 were unexposed) for second trimester and 3380 children consisted of 1583 discordant pairs of differentially exposed siblings (1594 were exposed and 1786 were unexposed) for third trimester.

For prescription with days' supply, there were 22146 children consisted of 10153 discordant pairs of differentially exposed siblings (10246 were exposed and 11900 were unexposed) for first trimester, 3954 children consisted of 1987 discordant pairs of differentially exposed siblings (1802 were exposed and 2152 were unexposed) for second trimester and 3108 children consisted of 1321 discordant pairs of differentially exposed siblings (1208 were exposed and 1900 were unexposed) for third trimester

© 2022 Chen VCH et al. *JAMA Network Open.*

eTable 3. Associations of Neurodevelopmental Disorders With Paternal BZD Dispensations During the First, Second, and Third Trimester of Pregnancy Using Population-Wide and Sibling Comparison Models

	Models	
	Adjustment Model[a]	Sibling comparison[b]
Period	HR (95% CI)	HR (95% CI)
First Trimester		
Autism spectrum disorder	0.94 (0.87-1.02)	1.07 (0.84-1.35)
Attention-deficit/hyperactivity disorder	1.03 (1.00-1.08)	1.02 (0.93-1.13)
Second Trimester		
Autism spectrum disorder	0.93 (0.85-1.00)	1.06 (0.84-1.34)
Attention-deficit/hyperactivity disorder	1.03 (1.00-1.08)	1.00 (0.91-1.11)
Third Trimester		
Autism spectrum disorder	1.04 (1.00-1.08)	1.31 (1.02-1.69)
Attention-deficit/hyperactivity disorder	1.04 (1.00-1.08)	0.98 (0.88-1.08)

Abbreviations: CI, confidence interval, HR, hazard ratio.

[a]Adjusted model controlled for year of birth, parity, gestational age, low-income family status, maternal and paternal nationality, paternal and maternal age at child birth, paternal and maternal history of mental disorders, smoking during pregnancy, and maternal opioid drug use.

[b]Sibling comparison controlled for year of birth, parity, gestational age, paternal and maternal age at child birth, paternal and maternal history of mental disorders, maternal smoking during pregnancy, and maternal opioid drug use.

For analysis of adjusted model, there were 64570, 64974, and 66216 BZD-exposures during the first, second and third trimester, respectively.

For analysis of sibling comparison, there were 50941 children consisted of 23961 discordant pairs of differentially exposed siblings (24350 were exposed and 26591 were unexposed) for first trimester, 50830 children consisted of 23918 discordant pairs of differentially exposed siblings (24315 were exposed and 26515 were unexposed) for second trimester and 51646 children consisted of 24302 discordant pairs of differentially exposed siblings (24676 were exposed and 26970 were unexposed) for third trimester.

© 2022 Chen VCH et al. *JAMA Network Open.*

eTable 4. Descriptive Statistics for Sample by Exposure to BZD Before Pregnancy

Variable	(n =65767)
Child variable	
Parity	
1	42316 (64.3)
2	20250 (30.8)
≥ 3	3201 (4.9)
Low-income family	4720 (7.2)
Gestational age, week	38.6±2.1
Child age, year	8.0±3.2
Child diagnosis	
Autism spectrum disorder	714 (1.1)
Attention-deficit/hyperactivity disorder	3281 (5.0)
Pregnancy Covariate	
Maternal Covariate	
Age at birth, year	
<20	581 (0.9)
20-24	7032 (10.7)
25-29	21371 (32.5)
30-34	25233 (38.4)
35-39	10014 (15.2)
≥40	1536 (2.3)
Taiwanese nationality	21647 (32.9)
Opioid drug use	5453 (8.3)
Smoking during pregnancy	32 (0.0)
Any mental disorders	17700 (26.9)
Paternal Covariate	
<20	93 (0.1)
20-24	2633 (4.0)
25-29	14098 (21.4)

© 2022 Chen VCH et al. *JAMA Network Open.*

30-34	25584 (38.9)
35-39	16336 (24.8)
≥40	7023 (10.7)
Taiwanese nationality	20199 (30.7)
Any mental disorders	7306 (11.1)

Abbreviations: BZD, benzodiazepine.

© 2022 Chen VCH et al. *JAMA Network Open*.

eTable 5. Associations of Neurodevelopmental Disorders With Maternal Benzodiazepine Exposures During the First, Second, and Third Trimester of Pregnancy, Compared With Exposure Before Pregnancy Using Population-Wide and Sibling Comparison Models

	Adjustment Model[a]	Sibling comparison[b]
Period	HR (95% CI)	HR (95% CI)
First Trimester		
Autism spectrum disorder	1.02 (0.91-1.14)	0.54 (0.17-1.71)
Attention-deficit/hyperactivity disorder	0.97 (0.92-1.02)	0.81 (0.56-1.16)
Second Trimester		
Autism spectrum disorder	1.00 (0.87-1.16)	2.71 (0.33-22.60)
Attention-deficit/hyperactivity disorder	0.99 (0.92-1.06)	0.71 (0.42-1.18)
Third Trimester		
Autism spectrum disorder	1.15 (0.90-1.46)	-[c]
Attention-deficit/hyperactivity disorder	0.97 (0.86-1.09)	0.46 (0.18-1.20)

Benzodiazepine exposures before pregnancy servers as the reference group.

Abbreviations: CI, confidence interval, HR, hazard ratio.

[a]Adjusted model controlled for year of birth, parity, gestational age, low-income family status, maternal and paternal nationality, paternal and maternal age at child birth, paternal and maternal history of mental disorders, smoking during pregnancy, and maternal opioid drug use.

[b]Sibling comparison controlled for year of birth, parity, gestational age, paternal and maternal age at child birth, paternal and maternal history of mental disorders, maternal smoking during pregnancy, and maternal opioid drug use.

[C]Analysis can not be estimated because of the insufficient of case number.

For analysis of sibling comparison, there were 4129 children consisted of 2043 discordant pairs of differentially exposed siblings (2060 were exposed and 2069 were unexposed) for first trimester, 2820 children consisted of 1392 discordant pairs of differentially exposed siblings (1406 were exposed and 1414 were unexposed) for second trimester. and 1010 children consisted of 495 discordant pairs of differentially exposed siblings (499 were exposed and 511 were unexposed) for third trimester.

© 2022 Chen VCH et al. *JAMA Network Open.*
